# Effect of Linker Sequence on the Interdomain Dynamics of Self-Sufficient CYP116B5-SOX Chimeras

**DOI:** 10.1021/acsomega.6c07054

**Published:** 2026-09-01

**Authors:** Daniele Giuriato, Lorenzo Gorga, Silvia Castrignanò, Danilo Correddu, Gianluca Catucci, Gianfranco Gilardi

**Affiliations:** Department of Life Sciences and Systems Biology, 9314 University of Torino, via Accademia Albertina 13, Torino 10123, Italy

## Abstract

In this work, the design of CYP116B5-SOX­(PPII) is presented. This is an artificial self-sufficient enzyme in which the P450 116B5 peroxygenase and the H_2_O_2_-generating sarcosine oxidase (SOX) are linked together in a fusion complex. A polyproline type II helix (PPII) linker was used to connect the two enzymes, obtaining a rigid fusion protein. CYP116B5-SOX­(PPII) displayed 10-fold increased activity in terms of *p*-nitrophenol oxidation (*k*_cat_: 32.1 ± 0.5 min^–1^; *K*_M_: 150 ± 15 μM) compared to the isolated heme domain CYP116B5-hd, and more than 2-fold higher activity compared to CYP116B5-SOX—a fusion enzyme in which the two domains are passively linked by a flexible polyglycine sequence. The stability of the three CYP116B5 variants was investigated by differential scanning calorimetry. The data revealed the higher folding cooperativity of the P450 domain within the rigid fusion system, as well as the stabilization of the SOX domain compared to CYP116B5-SOX. The catalytic stability of the fusion enzymes was confirmed by testing the single domains’ residual activity after thermal denaturation. The interdomain plasticity of the two fusion enzymes was analyzed by H/D exchange kinetics experiments through ATR-FTIR spectroscopy. The results revealed that the flexible CYP116B5-SOX exhibits a globally lower protein surface accessibility to water compared to the rigid CYP116B5-SOX­(PPII), suggesting that the formation of interdomain contacts is disadvantageous for this fusion couple. CYP116B5 shows some activity toward styrene oxidation into styrene oxide and phenylacetaldehyde (*K*_M_: 9.0 ± 2.9 mM, *k*_cat_: 1.9 ± 0.3 min^–1^). Interestingly, *E. coli* cells expressing CYP116B5-SOX­(PPII) show 56% styrene conversion when hydrogen peroxide is produced in situ by SOX, whereas only 13% conversion is reached when hydrogen peroxide is directly added in solution. Overall, this work shows that the stability and catalytic efficiency of the artificial chimera are intimately associated with the quaternary assembly of the construct, unveiling the crucial role of the fusion design for the extensive exploitation of P450 biocatalysis.

## Graphical abstract

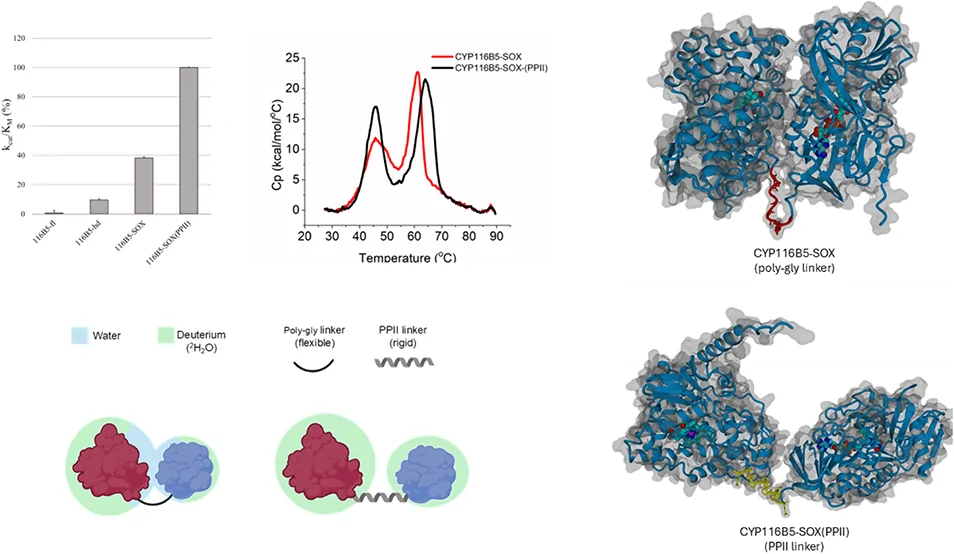

## 1 Introduction

The design of fusion enzymes by end-to-end genetic fusion is the most commonly used strategy to engineer multienzyme systems.^1,2^ By the *Molecular Lego* approach, the fusion of two genes that natively encode independent proteins can be achieved. Two or more redox/catalytic partners can be fused into a single polypeptide, generating artificial, catalytically self-sufficient enzymes.^3–6^ To date, the design of fusion enzymes is still primarily performed via a trial-and-error approach.^7^ The choice of the enzyme couple and the fusion-linker structure is empirical, and the fusion-domain quaternary assembly is difficult to predict.^1,8–10^ Recently, our laboratory discovered and biochemically characterized the drug-metabolizing CYP116B5 from *Acinetobacter radioresistens*.^11–13^ This cytochrome is natively a self-sufficient class VII P450, being fused to its FMN/2Fe2S-containing reductase. Interestingly, the peroxygenase activity of CYP116B5-hd (isolated heme domain) was found to be more efficient than the monooxygenase activity of the full-length form (CYP116B5-fl).^14^ In order to control the H_2_O_2_ supply and avoid its accumulation in solution, a fusion enzyme, called CYP116B5-SOX, was designed.^15^ This is a single-polypeptide fusion complex in which the peroxygenase activity of CYP116B5 is sustained by sarcosine oxidase (SOX) from *Bacillus* sp. *B-0618*, an H_2_O_2_-producing FAD-binding enzyme that oxidizes sarcosine into glycine and formaldehyde.^16–18^ Although the two enzymes were only passively connected using a polyglycine sequence, the fusion was shown to beneficially influence the catalytic efficiency of CYP116B5-SOX compared to both CYP116B5-hd and CYP116B5-fl.^14^ The exploitation of fusion enzyme catalysis in a whole-cell system was only possible due to the controlled in situ production of hydrogen peroxide provided by SOX.^15^ In this work, the CYP116B5 and SOX fusion design is further challenged by linking the two enzymes through a polyproline type II helix-forming sequence,^19–21^ obtaining the new CYP116B5-SOX­(PPII). The PPII linker is intrinsically rigid and introduces a structural constraint in the fusion enzyme assembly compared with the former construct. Previous studies showed that the use of rigid linkers minimizes fusion enzyme conformational changes over time, potentially benefiting protein stability and efficiency.^22,23^ Herein, the rigid CYP116B5-SOX­(PPII) self-sufficient activity is compared to both the flexible CYP116B5-SOX fusion protein and the isolated CYP116B5-hd. The stability in solution of the three CYP116B5 proteins was tested by differential scanning calorimetry and by measuring residual activity after thermal denaturation. The interdomain dynamics of the two fusion enzymes were analyzed by H/D exchange kinetics through ATR-FTIR spectroscopy (attenuated total reflectance-Fourier transform infrared spectroscopy). Finally, the CYP116B5-SOX­(PPII) system is exploited in *E. coli* cells to oxidize styrene, a CYP116B5 substrate herein identified through the screening of a library of alkyl- and aryl-containing compounds.

## 2 Results

### 2.1 Protein Expression and Purification

Starting from the sequence of the chimeric enzyme CYP116B5-SOX, a new construct was engineered by switching the domain-linker sequence within the expression vector pET-28a­(CYP116B5-SOX). The sequence coding for GPGGGGGGGPG between the CYP116B5 domain and the MSOX was substituted with a sequence coding for EPPPPLPPPPE. This polyproline type II helix-forming linker (PPII) is designed to increase the rigidity of the fusion enzyme, thus obtaining the new chimera CYP116B5-SOX­(PPII). The recombinant protein was expressed using *E. coli* as the host organism, following a similar protocol as previously reported for CYP116B5-SOX.^15^ The protein was purified from the cell-free extract by nickel-ion affinity chromatography, taking advantage of the 6xHis-tag at the C-terminus of the enzyme, followed by size-exclusion chromatography. The amount of protein at the end of the purification process was quantified on the basis of UV–vis absorption at 450 nm, relative to the ferrous-CO form of the enzyme. The recovery yield of CYP116B5-SOX­(PPII) was 32 mg of pure protein per liter of cell culture, a result three times higher than that obtained for CYP116B5-SOX (10.62 mg L^–1^).

### 2.2 Catalytic Efficiency

The peroxygenase activity of CYP116B5-SOX­(PPII) in the presence of excess concentration of sarcosine was evaluated by extrapolating the kinetic parameters for para-nitrophenol conversion into para-nitrocatechol using the typical Michaelis–Menten model. The *k*_cat_ of CYP116B5-SOX­(PPII) for *p*-NP conversion is 32.0 ± 0.5 min^–1^ (Figure 1, Panel A), nearly 12 times higher than that reported for CYP116B5-hd (2.7 ± 0.1 min^–1^),^12,13^ and more than 1.5 times that of the flexible fusion enzyme CYP116B5-SOX (20.1 ± 0.6 min).^15^ The affinity for *p*-NP (*K*_M_) slightly changes among the three enzyme systems: 150 ± 15 μM for CYP116B5-SOX­(PPII), 230 ± 30 μM for CYP116B5-SOX, and 128 ± 30 μM for CYP116B5-hd, indicating a higher affinity for substrate binding of the rigid construct for *p*-NP compared to the flexible one, and more similar to the isolated heme domain. The data suggest a higher exposure of the binding site of CYP116B5 in the rigid construct, suggesting better fusion-domain separation within the CYP116B5-SOX­(PPII) structure. When *k*_cat_/*K*_M_ is compared, CYP116B5-SOX and CYP116B5-hd (isolated heme domain) respectively display 38% and 9% of CYP116B5-SOX (PPII)’s activity (Figure 1, Panel B). The activity of CYP116B5-fl^14^ is about 4% of the self-sufficient CYP116B5-SOX­(PPII). The beneficial effects of in situ H_2_O_2_ transfer in self-sufficient peroxidases have been described for CYP116B5-SOX^14,15^ and for similar fusion enzymes.^24–27^ Controlled hydrogen peroxide production has been shown to be fundamental in preventing the rapid oxidative inactivation of the enzyme, which commonly occurs in P450s.^28^ Our data indicate that the advantageous local delivery of hydrogen peroxide is preserved in CYP116B5-SOX­(PPII) following the linker switch, supporting the proposed mechanism.

**Figure 1:**
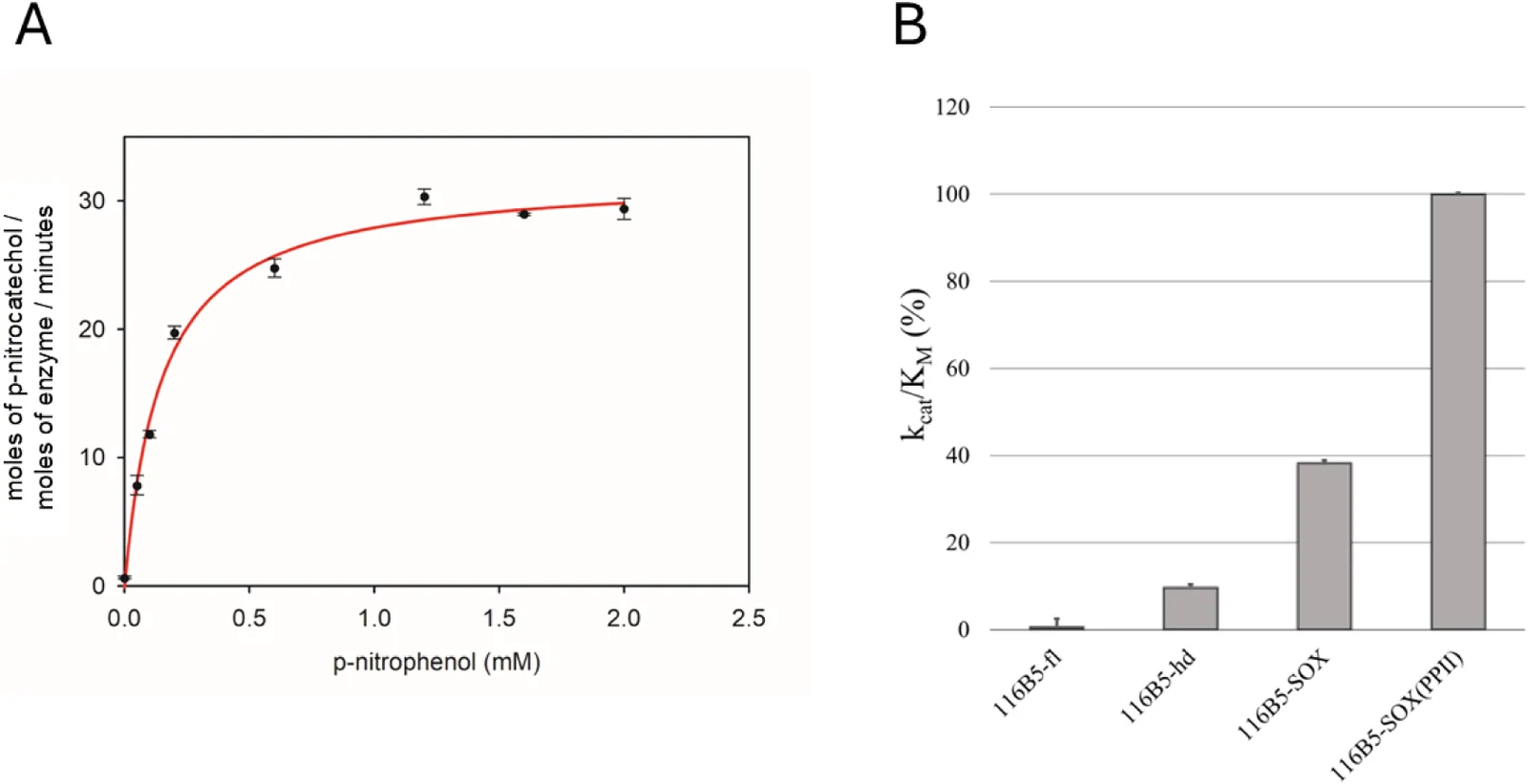
Kinetic parameters of CYP116B5 variants. (A) Michaelis–Menten curve fitting of the rate of conversion of pNP into pNC by CYP116B5-SOX­(PPII) catalysis driven by sarcosine. (B) Catalytic efficiency comparison of the CYP116B5 variants. The specificity constant of CYP116B5-SOX­(PPII) was used as a benchmark to compare the relative activity of the different P450 isoforms. Data represent the average of three replicates. Error bars represent standard deviation; error bars are not visible when they are smaller than the data point symbols.

### 2.3 Differential Scanning Calorimetry

The denaturation process of CYP116B5-SOX and CYP116B5-SOX­(PPII) was investigated by differential scanning calorimetry. The unfolding transition of the two fusion systems was monitored using a temperature ramp ranging from 25 to 90 °C at a scan rate of 90 °C/h.^3,29^ Figure 2 shows the thermograms of CYP116B5-SOX and CYP116B5-SOX­(PPII). The experimental curves were fitted using a non-two-state denaturation model to extrapolate the thermodynamic parameters, which are summarized in Table 1. Two endothermal peaks were observed for both fusion systems, which are the signals associated with the denaturation of the two fusion domains.^15^ Overall, the data show that the unfolding behavior of the two fusion domains is conserved in the two constructs, although some significant differences can be observed. While the melting temperature (*T*_M_) of the CYP116B5 domain is similar between the two fusion systems, the SOX domain shows an increase in the melting temperature of +3.2 °C in CYP116B5-SOX­(PPII). The peak width at half-maximum (*T*_1/2_) of the CYP116B5 domain was found to be lower in CYP116B5-SOX­(PPII) (6.3 ± 0.1 °C) compared to CYP116B5-SOX (10.9 ± 0.1 °C), implying that the unfolding transition of the P450 takes place over a narrower temperature range for the rigid construct. This phenomenon can be associated with more cooperative folding of the P450 domain within the rigid construct, with a positive effect on its stability in solution. The increased *T*_M_ of SOX and the decreased *T*_1/2_ of CYP116B5 result in the overall better separation of the unfolding of the two fusion domains that can be observed in CYP116B5-SOX­(PPII) (Figure 2). The enthalpy values of CYP116B5-SOX­(PPII) and CYP116B5-SOX extrapolated by curve fitting are 330 and 299 kcal/mol, respectively, implying a slightly higher total energy barrier to unfolding of the rigid construct. In general, calorimetric analysis indicates that while the folding of the two single domains is conserved in the two constructs, the rigid polyproline helix exerts a positive effect on the fusion system, ensuring both distancing and rigidity of the two domains.

**Figure 2:**
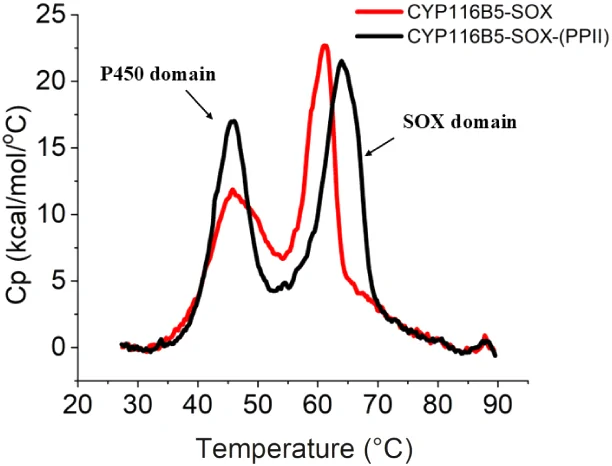
Denaturation profiles of CYP116B5-SOX­(PPII) (black curve) and CYP116B5-SOX (red curve) obtained by differential scanning calorimetry experiments. Thermograms were obtained by applying a temperature gradient of 25–90 °C with a scan rate of 90 °C/h after 10 min of prescan equilibration; the same buffer was also used for the reference scan. The protein sample was suspended at a final concentration of 0.6 mg/mL in 50 mM KPi at pH 6.8 supplemented with 1% glycerol.

**Table 1:** Thermodynamic Parameters Associated with CYP116B5-SOX­(PPII) and CYP116B5-SOX Denaturation Profiles

	CYP116B5-SOX(PPII)	CYP116B5-SOX
*T*_M_1	45.8 ± 0.1 °C	46.2 ± 0.1 °C
Δ*H*1	141.8 ± 2.2 kcal/mol	152.5 ± 3.7 kcal/mol
*T*_1/2_1	6.3 ± 0.1 °C	10.9 ± 0.1 °C
*T*_M_2	63.8 ± 0.1 °C	60.6 ± 0.1 °C
Δ*H*2	188.9 ± 2.2 kcal/mol	147.0 ± 2.9 kcal/mol
*T*_1/2_2	6.4 ± 0.1 °C	5.0 ± 0.1 °C

### 2.4 Enzyme Stability

In order to test the catalytic stability of the CYP116B5 variants, the residual *p*-nitrocatechol production by CYP116B5-hd, CYP116B5-SOX, and CYP116B5-SOX­(PPII) was measured after thermal exposure of the proteins to increasing temperatures. The peroxygenase activity of CYP116B5-SOX and CYP116B5-SOX­(PPII) was sustained by an excess concentration of sarcosine, whereas that of CYP116B5-hd was sustained by an excess of H_2_O_2_. Results were fitted to a four-parameter Hill-like equation. The *T*_50_ parameter and the Hill constant (*b*), which describes the cooperativity of the unfolding transition, were used to define the temperature-dependent stability of the three enzymes^30^ (Figure 3, Table 2). All the enzyme variants show the typical cooperative single-step sigmoidal curve^31^ (Figure 3). By comparing the extrapolated *T*_50_ values of the three systems, no significant differences were observed (Table 2). On the other hand, CYP116B5-SOX­(PPII) showed an almost doubled Hill constant (*b*: 50.8 ± 1.7) compared to CYP116B5-SOX (*b*: 26.2 ± 1.6), and both fusion enzymes showed higher Hill constants compared to the isolated CYP116B5-hd (*b*: 21.0 ± 1.7). The data suggest higher unfolding cooperativity of the rigid construct, which is commonly associated with an increase in protein stability in solution. Together with the DSC analysis results, these data suggest that enhanced P450-domain cooperativity within CYP116B5-SOX­(PPII) positively affects the overall catalytic stability of the construct.

**Figure 3:**
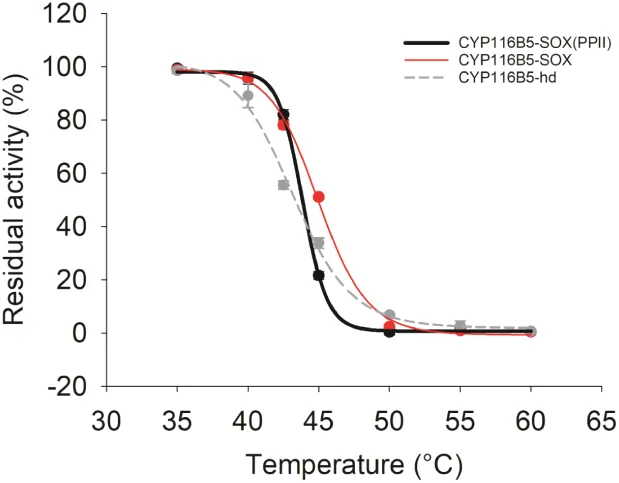
Residual pNC production of CYP116B5-SOX­(PPII) (black dots, thick black line), CYP116B5-SOX (red dots, red line), and CYP116B5-hd (gray dots, dashed gray line) after thermal denaturation at the indicated temperatures. All reactions were performed in 50 mM KPi buffer at pH 6.8 supplemented with 1.2 mM pNP and 250 mM sarcosine for fusion enzymes or 1 mM hydrogen peroxide for CYP116B5-hd. Each enzyme was used at a final concentration of 0.25 μM. Lines represent Hill-like equation fitting. Error bars represent the standard deviation of at least three replicates and are not visible when shorter than the symbol point.

**Table 2:** Hill’s Equation Parameters Extrapolated for Activity Decay at Scalar Temperatures of the Three P450 116B5 Variants^a^

	CYP116B5SOX(PPII)	CYP116B5SOX	CYP116B5hd
*T*_50_ (°C)	43.9 ± 0.1	45.0 ± 0.1	43.2 ± 0.2
Hill constant *b*	50.8 ± 1.7^b^	26.2 ±1.6^c^	21.0 ± 1.7

a The experiment was performed by measuring residual pNC production after thermal denaturation. Statistical analysis was performed by Welch’s *t*-test.

b *P* < 0.0001.

c *P* < 0.02 compared to CYP116B5-hd.

### 2.5 Sarcosine Oxidase Activity

In the systems studied in this work, SOX produces hydrogen peroxide to sustain the peroxygenase activity of CYP116B5. To evaluate the effect of the fusion linker on SOX activity, the production of hydrogen peroxide was monitored by measuring the formation of ABTS•^+^ during a SOX-horseradish peroxidase coupled reaction, as previously reported.^15,24,32^ The peroxide production was followed for 1200 s at the reference temperature of 30 °C in the presence of an excess of sarcosine^15,18,24^ (Figure 4, Panel A). CYP116B5-SOX displays a higher initial rate of hydrogen peroxide production, reaching 2589 ± 15 min^–1^ within the first 200 s of reaction, whereas the rate measured for CYP116B5-SOX­(PPII) was 1975 ± 37 min^–1^. More interestingly, CYP116B5-SOX (PPII) shows a steady hydrogen peroxide production over the entire experiment time course (1200 s), while CYP116B5-SOX displays an overall lower total turnover (Figure 4, Panel A). The data suggest that, despite a lower initial turnover rate, the stability of CYP116B5-SOX (PPII) during catalysis increased compared to CYP116B5-SOX. To evaluate the thermal stability of the two systems, the enzymes were incubated at increasing temperatures, and the residual activity of SOX was measured (Figure 4, Panel B). CYP116B5-SOX (PPII) displays a higher residual activity compared to CYP116B5-SOX (Figure 4, Panel B). The data indicate the overall higher stability of SOX within the rigid fusion system, suggesting the beneficial effect of the PPII linker on the SOX enzyme.

**Figure 4:**
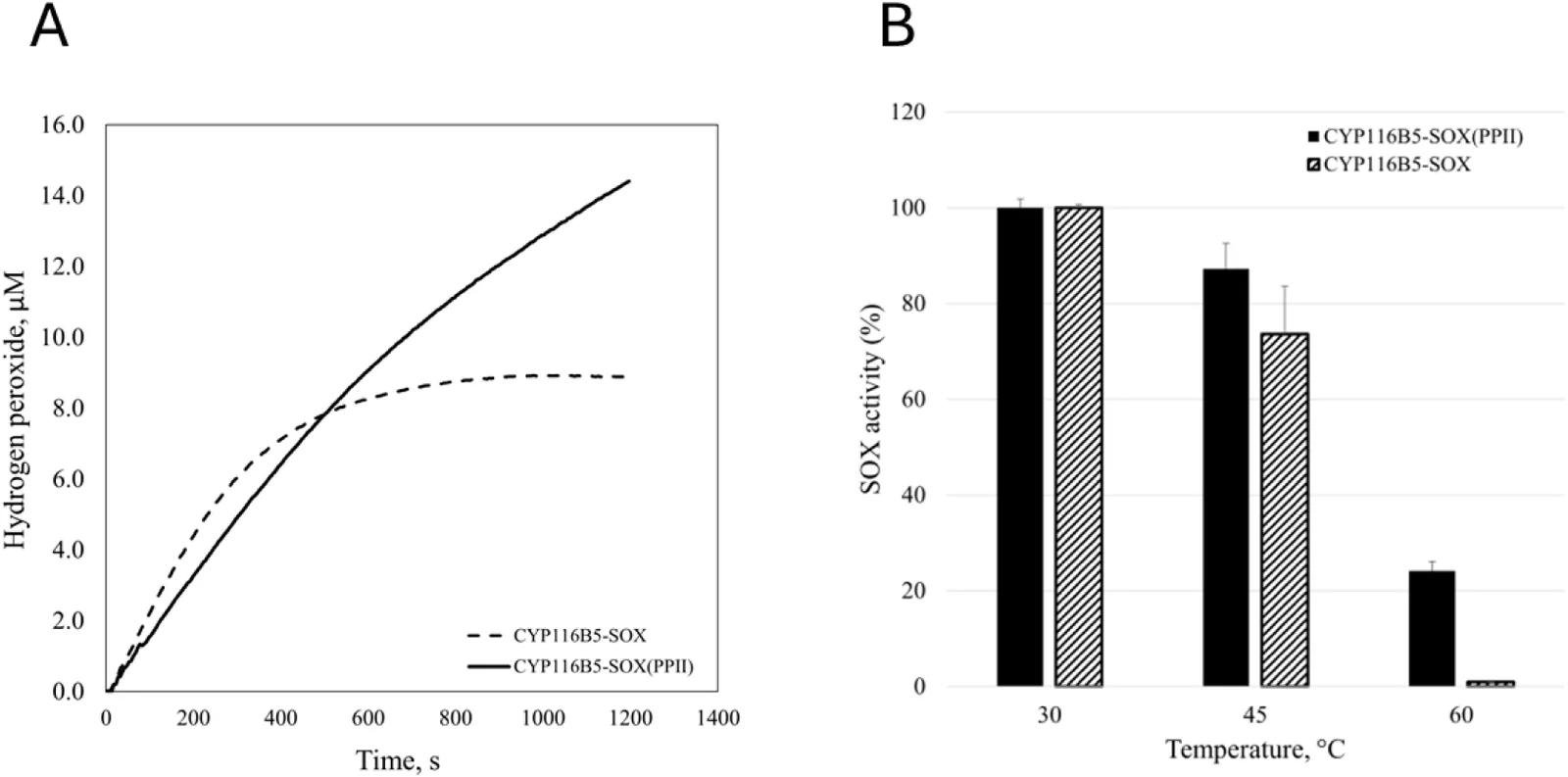
(A) Hydrogen peroxide production time course by CYP116B5-SOX (dotted line) and CYP116B5-SOX­(PPII) (solid line) in the presence of sarcosine. Each curve is the average of three replicates. (B) Residual activity of the SOX domain of CYP116B5-SOX (black solid bars) and CYP116B5-SOX­(PPII) (dashed bars) after thermal denaturation at the indicated temperatures. The activity at 30 °C was used as the benchmark to calculate relative activity in all the other conditions. All reactions were performed in 50 mM KPi buffer at pH 6.8 supplemented with 5 mM sarcosine. Each enzyme was used at a final concentration of 3 nM. Error bars represent the standard deviation of at least three replicates.

### 2.6 H/D Exchange Kinetics

In light of the significant changes in the activity and stability of the two constructs, we hypothesized that linker rigidity might influence the quaternary assembly of the fusion enzyme. To explore the structural plasticity of the differentially linked fusion domains, H/D exchange measurements by Attenuated Total Reflectance—Fourier Transform Infrared Spectroscopy (ATR-FTIR) experiments were performed.^33–36^ The infrared spectral variations detected during H/D exchange in protein samples are predominantly associated with peptide -bond vibrations. Specifically, the Amide II band (∼1550 cm^–1^) is sensitive to the degree of deuteration in the environment, since it mainly results from the in-plane N–H bending coupled with C–N stretching within the peptide bond. On the other hand, the Amide I band (∼1650 cm–^1^) is minimally influenced by the substitution of hydrogen with deuterium, since it is primarily due to the stretching vibrations of the peptide C=O group. In this experiment, the protein sample in aqueous solution is progressively exposed to deuterated water vapor (^2^H_2_O). As H/D exchange progresses, N–H bonds are replaced by N–D bonds, leading to a proportional shift of the Amide II band absorption from ∼1550 cm^–1^ (N–H) to ∼1450 cm–1 (N–D), in accordance with the extent of protein proton exchange (Figure 5). By plotting the relative Amide II signal as a function of exposure time to deuterated water, the protein accessibility to the solvent can be studied (Figure 6). For both CYP116B5-SOX­(PPII) and CYP116B5-SOX, three populations of protons with different exchange rates can be distinguished. In keeping with published literature,^10,31^ the first population of protons to be exchanged (10 min) was assigned to the fully exposed peptide bonds at the surface of the protein. The second proton population, which is exchanged at a longer time, was assigned to the less exposed peptide bonds at the protein surface. Finally, the third population of nonexchangeable protons was assigned to the fraction of buried peptide bonds not exposed to the solvent. H/D exchange kinetics were studied by fitting the curve of the unexchanged proton fraction to the sum of two exponential equations, resulting in the rate constants *k*_1_ and *k*_2_, respectively, for the first two populations, and in the parameter *F*_0_ for the nonexchangeable fraction (Table 3). Statistical analysis showed that no significant differences were observed in the exchange rate of the fully exposed protons in the two constructs (Table 3, *k*_1_). Most interestingly, CYP116B5-SOX­(PPII) shows a significant increase in the rate of exchange of the less exposed protons (*k*_2_: 0.556 ± 0.135) compared to CYP116B5-SOX (*k*_2_: 0.273 ± 0.098), as well as a lower *F*_0_ (24.2 ± 1.8% versus 29.0 ± 3.3%, respectively) (Table 3). Taken together, the data indicate a globally higher protein-surface accessibility to the solvent of the rigid construct. In the case of two proteins that differ only in the linker sequence, the change in surface exposure to the solvent can be interpreted in terms of fusion-domain plasticity, in line with previous studies.^10,33^ A representative scheme is shown in Figure 8, Panel A. In knowledge of published literature,^7^ the flexible poly-Gly linker was designed to not restrict the spontaneous reciprocal movements of the two fusion domains. On the other hand, the highly rigid PPII linker stabilizes the mutual positions of the two fusion domains, keeping a fixed distance between them. It can be inferred that the conformational flexibility of CYP116B5-SOX favors the formation of more interdomain contacts, resulting in the overall lower exposure of the protein surface to the solvent. Instead, the rigid conformation of CYP116B5-SOX­(PPII) minimizes the number of interdomain interactions, maximizing surface exposure. Interestingly, the data presented in this work show that the formation of more intramolecular interactions in the chimera is disadvantageous for the stability and activity of P450 116B5 and SOX. Figure 8 Panel B shows the AlphaFold-predicted models of CYP116B5-SOX and CYP116B5-SOX­(PPII) with the highest achievable accuracy (pTM of 0.55 and 0.55, ipTM of 0.61 and 0.60 for CYP116B5-SOX and CYP116B5-SOX­(PPII), respectively).^37,38^ The models provide a plausible representation of the subunits’ positions in the two constructs, showing the linker-dependent change in protein-surface exposure, as experimentally measured herein.

**Figure 5:**
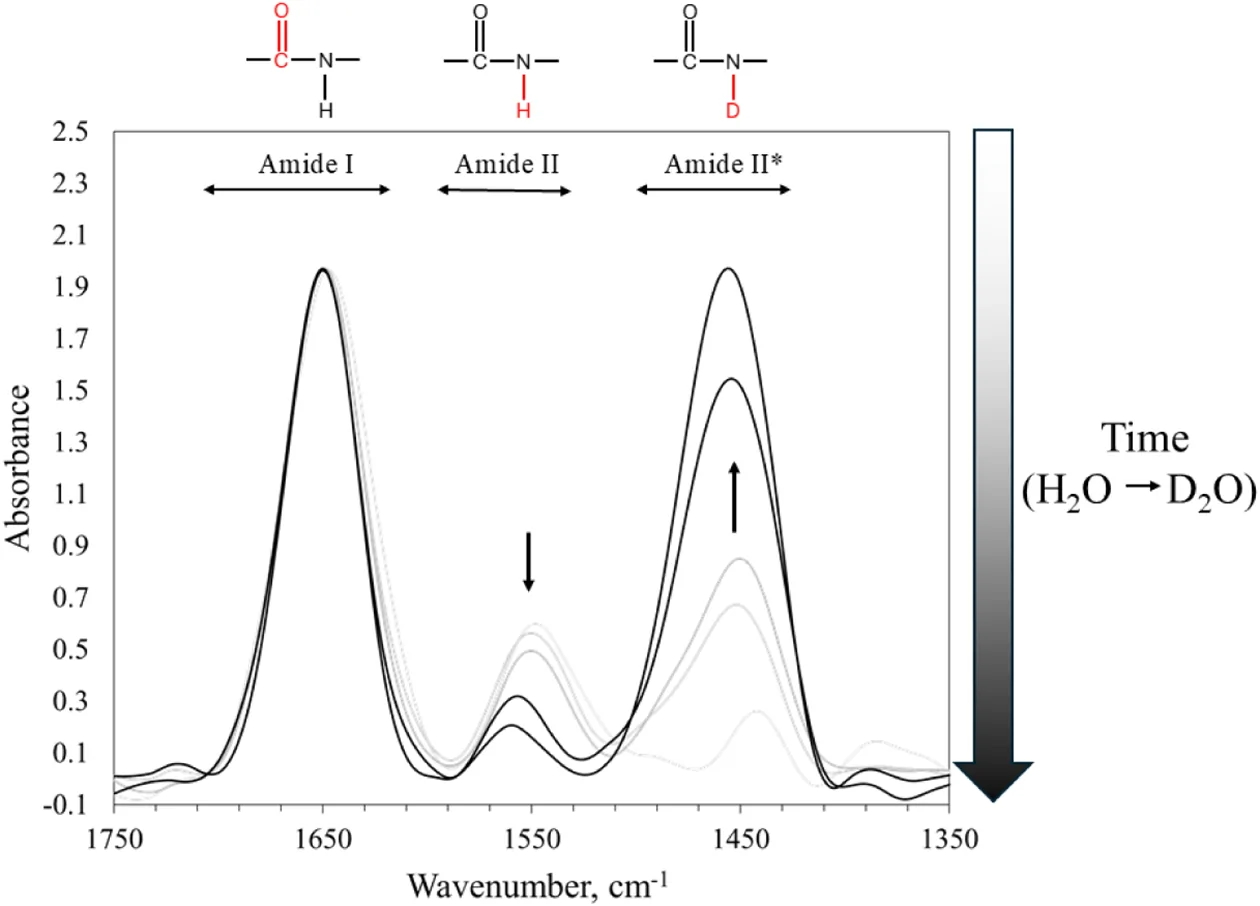
FTIR spectral transition of CYP116B5-SOX­(PPII) during H/D exchange. Spectra were recorded as a function of time after connecting the sample cell to a D_2_O-vapor-saturated nitrogen flow. The spectra are normalized to the Amide I signal. The typical frequency ranges of the amide bands and the corresponding bond interested are displayed.

**Figure 6:**
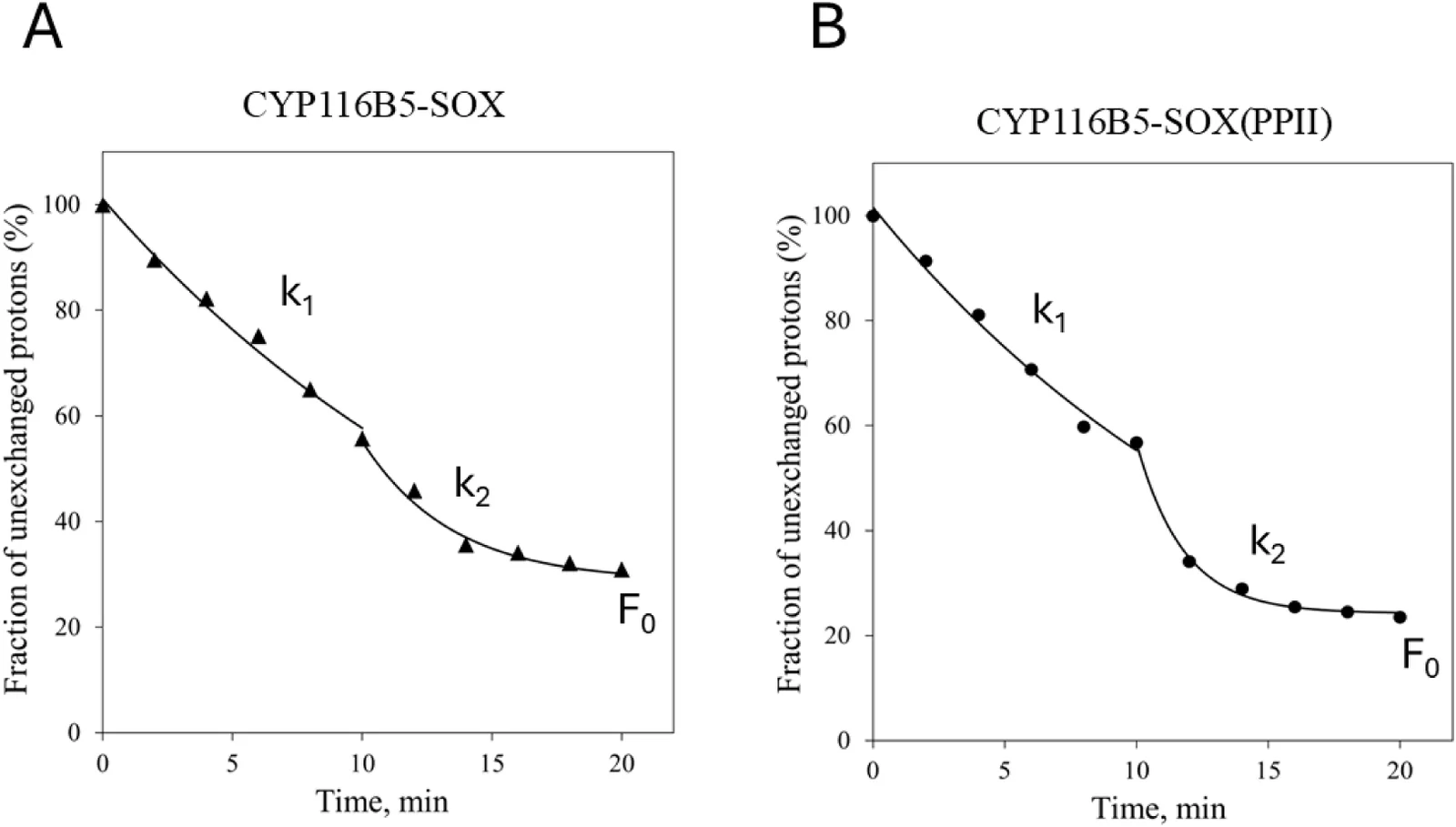
Normalized fractions of unexchanged amide protons as a function of ^2^H_2_O exposure time measured for CYP116B5-SOX (A) and CYP116B5-SOX­(PPII) (B). Solid lines represent the best fit to the sum of two single-exponential curves of the data points.

**Table 3:** Fitted H/D Exchange Kinetics Parameters^a^

	CYP116B5-SOX	CYP116B5-SOX(PPII)
*k*_1_ (min^–1^)	0.056 ± 0.004	0.060 ± 0.005
*k*_2_ (min^–1^)	0.273 ± 0.098	0.556 ± 0.135^b^
*F*_0_ (%)	29.0 ± 3.3	24.2 ± 1.8^b^

a Statistical analysis was performed by Welch’s *t*-test.

b *P* < 0.05 compared to CYP116B5-SOX.

### 2.7 Conversion of Styrene

The CYP116B5-hd activity was tested toward a library of small alkyl- and aryl-containing compounds, including linalool, ethyl levulinate, indene, naphthalene, tetralin, S-limonene, R-limonene, and styrene. The enzyme was shown to produce two oxidation products from styrene: styrene oxide and phenylacetaldehyde, with a ratio 35:65 (Figure 7, Panel A). Interestingly, CYP116B5-SOX­(PPII) catalysis produced a low amount of a byproduct of styrene conversion–2-phenylpropanal (∼13%)—in addition to styrene oxide (71%) and phenylacetaldehyde (16%) (Figure 7, Panel A). A similar change in product specificity was observed for P450 SPα—a P450 that displays similar activity toward styrene—when fused with SOX to convert styrene.^21^ It was proved that the formation of 2-phenylpropanal yields from the addition of formaldehyde, produced by the SOX-mediated oxidation of sarcosine,^18^ to phenylacetaldehyde. Kinetic studies of styrene turnover by CYP116B5-SOX­(PPII) showed a *K*_M_ value of 9.0 ± 2.9 mM and a *k*_cat_ of 1.9 ± 0.3 min^–1^. Previously, it was shown that the self-sufficient catalysis of CYP116B5-SOX is fundamental to efficiently transfer hydrogen peroxide to the P450 and to increase the yield of tamoxifen production in transformed *E. coli* cells.^15^ To test the applicability of the system in study, CYP116B5-SOX­(PPII) was used in *E. coli* cell lysate to scale up styrene conversion. When hydrogen peroxide is added in bulk directly to the cell lysate, 13% of the initial concentration of styrene (1 mM) is converted into styrene oxide as the main product, with detectable traces of phenylacetaldehyde as a byproduct. Notably, the conversion of styrene reaches 56% when hydrogen peroxide is generated in situ by SOX activity and is driven by sarcosine (Figure 7, Panel B). The data confirm the enhanced performance of the P450 when its catalysis is associated with SOX compared to the exogenous addition of hydrogen peroxide to the cell batch, underscoring the crucial role of the H_2_O_2_ transfer efficiency.

**Figure 7:**
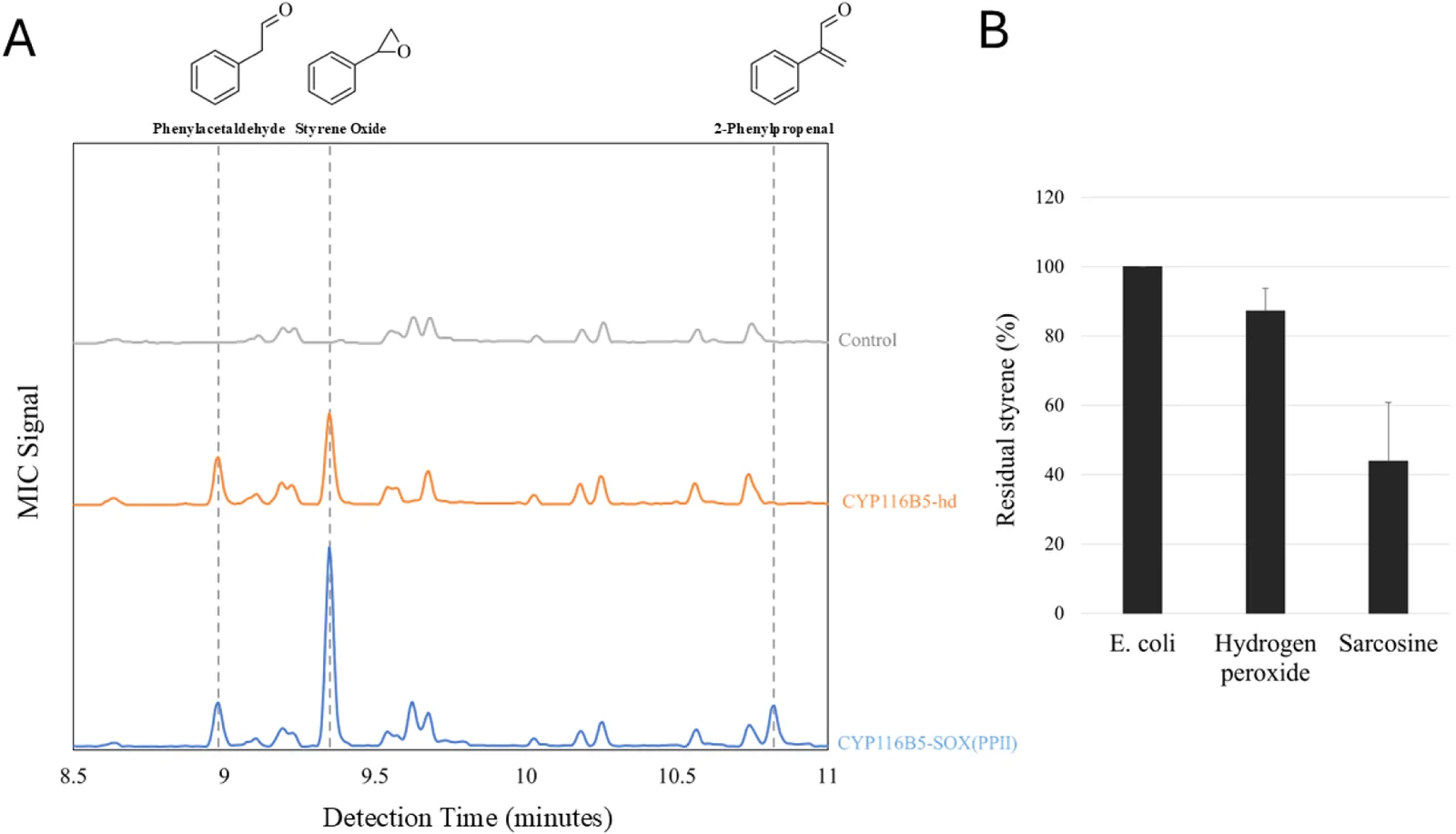
Styrene reaction analysis. (A) GC-MS signal detected by multiple-ion counting (*m*/*z*: 91, 104, 132) relative to the reaction of the purified CYP116B5 isolated heme domain (CYP116B5-hd) in the presence of H_2_O_2_ and CYP116B5-SOX­(PPII) in the presence of sarcosine. The control sample is obtained by mixing CYP116B5-SOX­(PPII) with styrene in the absence of H_2_O_2_ or sarcosine. (B) Styrene conversion by *E. coli* cells transformed with CYP116B5-SOX­(PPII) in the presence of hydrogen peroxide or sarcosine.

## 3 Discussion

Taken together, the data presented in this work show the advantage of using a rigid polyproline helix compared to a flexible polyglycine linker for the fusion of CYP116B5 and SOX. An improvement in the activity and stability of fusion enzymes upon the use of a polyproline linker has been reported for several constructs. Previously, it has been demonstrated that the activity of the Fpr/YkuN fusion construct could be tuned by linker engineering, with overall best performance using proline linkers compared to glycine linkers.^23^ In flavodoxin (FldA) and flavodoxin reductase (Fpr) fusion proteins, constructs carrying proline-rich linkers generally outperform their glycine-rich counterparts.^22^ The presence of a proline-rich linker resulted in higher activity of FDH in BM3-FDH fusion enzymes compared to shorter and more flexible linkers.^39^ The data presented in this work show an almost 2-fold increase in the self-sufficient peroxygenase activity of CYP116B5-SOX­(PPII) compared to CYP116B5-SOX in terms of catalytic efficiency for the conversion of *p*-nitrophenol into *p*-nitrocatechol. The enhanced substrate affinity (*K*_M_) of CYP116B5-SOX­(PPII) suggests greater exposure of the binding site of CYP116B5 in the rigid construct, indicating better separation of the fusion domains within the CYP116B5-SOX­(PPII) structure. DSC analysis showed that the unfolding behavior of the two single domains is globally conserved in the two constructs. Moreover, a positive effect on the folding cooperativity of the P450 and on the melting temperature of the SOX domains is observed in CYP116B5-SOX­(PPII). The effect of the polyproline helix linker on the P450 and SOX domains was further investigated by studying the catalytic stability of the differentially fused domains. The analysis confirmed the higher folding cooperativity of P450 116B5 and the enhanced thermal stability of SOX when they are fused together through a rigid linker. The interdomain plasticity of the two constructs was investigated by measuring the change in the rate of H/D exchange at the surface of the protein by ATR-FTIR analysis. The DSC analysis showed that the respective tertiary structures of the two fusion domains do not drastically change in the two constructs. Therefore, we interpreted the H/D exchange results in terms of the mutual positioning of the two fusion domains. The globally lower surface accessibility to solvent of CYP116B5-SOX compared to CYP116B5-SOX­(PPII) suggests the formation of more contacts between the fusion domains when they are linked by the flexible polyglycine sequence. The increase in the conformational rigidity of the fusion protein keeps a fixed distance between the two fusion domains, minimizing the nonproductive interactions between them, also in line with the substrate affinity data. AlphaFold was used to predict the structure of the two constructs (Figure 8, Panel B). The accuracy of the predicted relative positions of the subunits within the constructs is in the suboptimal range (ipTM 0.61 and 0.60 for CYP116B5-SOX and CYP116B5-SOX­(PPII), respectively),^37,38^ likely because of the intrinsically disordered nature of the linkers. Nevertheless, in light of the experimental data on the surface solvent exposure of the two constructs, we may assume that the two models are a likely representation of the CYP116B5-SOX and CYP116B5-SOX­(PPII) overall structure, useful for visualization purposes. Within the CYP116B5-SOX­(PPII) structure, the backbone dihedral torsion angles of the polyproline sequence fall within the range associated with a left-handed polyproline type II (PPII) helix (ϕ = −76° to −53°; ψ = 133° to 155°),^19,20,40^ showing the conformation adopted by the linker. Future studies will be dedicated to assessing the static and dynamic nature of the protein–protein interactions within the constructs. In particular, pulsed EPR spectroscopy coupled to protein spin labeling is envisioned as the best technique to further improve and experimentally validate the details of the proposed structural model. Similar to what was previously observed for SPα-SOX chimeras,^21^ the concerted activity of CYP116B5 and SOX shows a change in the product profile of styrene conversion compared to the isolated P450 activity. Most interestingly, artificially self-sufficient fusion enzymes show to be an effective strategy to scale up the peroxygenase activity of the P450 in bacterial cells, as shown by the 43% increase in styrene conversion of *E. coli* lysate compared to the direct addition of H_2_O_2_. In light of these results, we can conclude that the global effect on the catalytic efficiency and stability of CYP116B5-SOX, obtained by switching the fusion linker sequence, may be associated with the different conformational assembly and mutual domain positions in the fusion protein. Previous studies investigated how intramolecular protein interactions in multidomain proteins depend on linker architecture, experimentally measuring how physical and chemical properties of linkers affect the effective concentrations in fusion constructs.^41–43^ Interestingly, the data presented in this work show that the formation of protein–protein interactions between two independently evolved enzymes, which have not been selected to productively interact, may be disadvantageous for their stability and combined activity, further evidencing the importance of the linking strategy for fusion-protein engineering.

**Figure 8:**
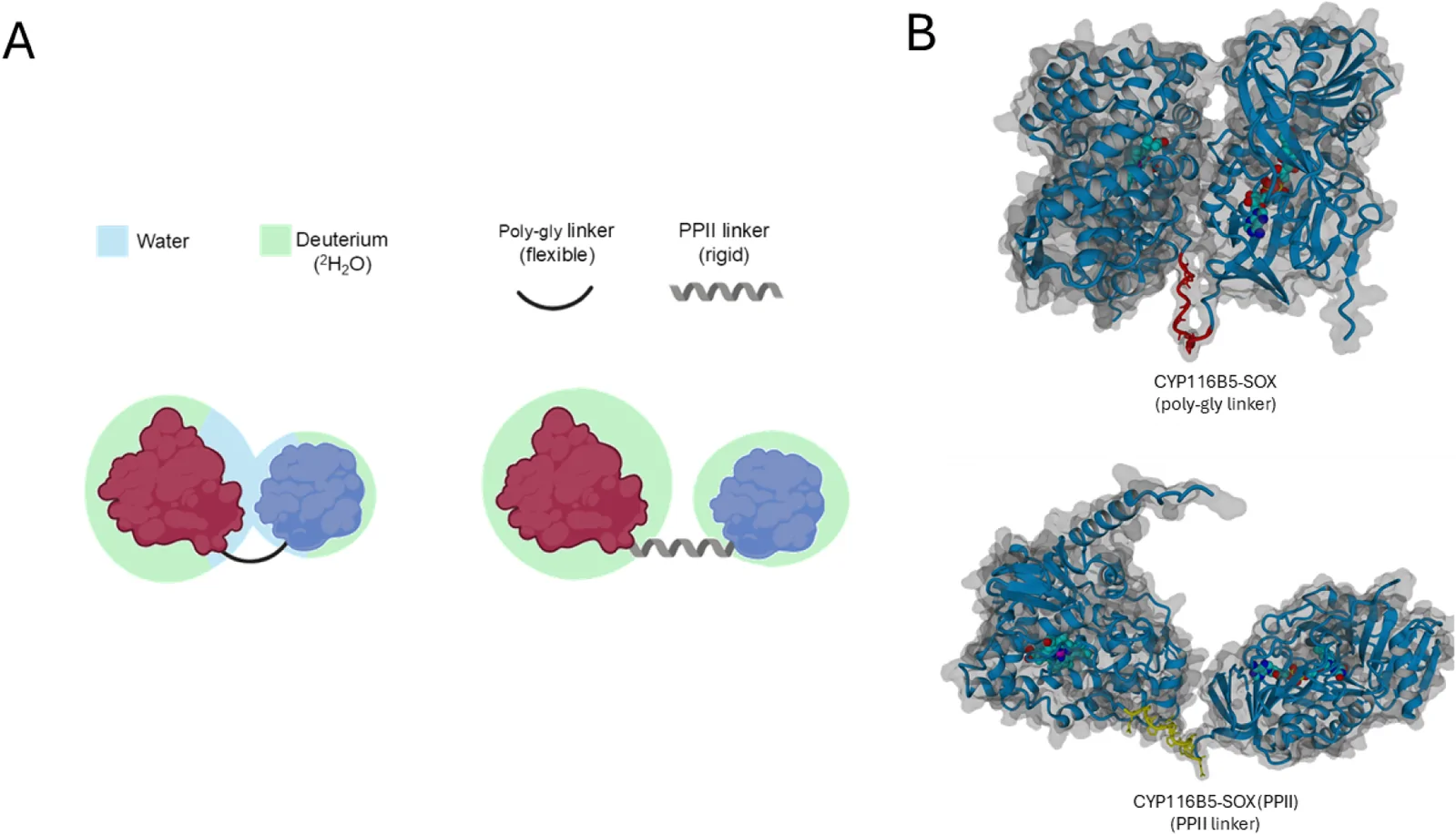
(A) Schematic representation of H/D-exchange results of differentially linked CYP116B5-SOX fusion proteins. Regions of the surface where hydrogen exchanges rapidly with deuterium are shown in green, and the less exposed regions where hydrogen exchange is slower or absent are shown in blue. (B) AlphaFold-predicted models of CYP116B5-SOX and CYP116B5-SOX­(PPII). The protein structure is represented as a ribbon and colored in blue, except for the linker sequence; the poly-Gly linker is colored in red and the PPII linker is colored in yellow, cofactors are represented as spheres, and the atomic molecular surface is shown in gray.

## 4 Methods

### 4.1 Enzyme Preparation

CYP116B5-SOX^15^ and CYP116B5-hd^12^ were prepared as previously described. CYP116B5-SOX­(PPII) was expressed using the same method reported for CYP116B5-SOX^14^ and purified using a similar method with some modifications. In brief, cells were harvested and lysed by sonication (5 × 30 s pulses) with a Misonix Ultrasonic Sonicator (Teltow, Germany) in a buffer containing 50 mM potassium phosphate (pH 6.8), 100 mM KCl, and 1% Triton X-100, supplemented with 1 mg/mL lysozyme, 0.1 mg/mL DNase I, and 1 mM PMSF. The lysate was ultracentrifuged at 180,000*g* for 50 min, and the resulting cell-free extract was loaded onto a pre-equilibrated 5 mL nickel-ion affinity column (His-trap HP, GE Healthcare) maintained at 25 °C. The column was washed with 50 mM potassium phosphate (pH 6.8) and then with 35 mM, 50 mM, and 65 mM imidazole. His-tagged proteins were eluted using a 75 mM imidazole. His-trap elution fractions were pooled and further purified by size-exclusion chromatography (HiLoad 16/600 Superdex 200 pg) equilibrated with 50 mM potassium phosphate buffer (pH 6.8) supplemented with 200 mM KCl.

### 4.2 Kinetic Parameters Evaluation

CYP116B5-SOX (PPII) was suspended in 50 mM KPi at pH 6.8 at a final concentration of 0.25 μM; the concentration of para-nitrophenol (*p*-NP) varied among different samples: 0, 0.05, 0.1, 0.2, 0.6, 1.2, 1.6, and 2 mM. These concentrations of *p*-NP were used to construct the Michaelis–Menten curve for the catalysis of CYP116B5-SOX­(PPII). The reaction was initiated by adding 250 mM sarcosine, and samples were incubated at 30 °C for 5 min. To terminate the reaction, 1% v/v trichloroacetic acid (TCA) was added to each sample, followed by centrifugation at 15,000*g* for 10 min to remove insoluble denatured enzyme. The concentration of para-nitrocatechol (p-NC) produced was evaluated by measuring absorbance at 515 nm (ε = 12.4 mM^–1^ cm^–1^) after adjusting the sample pH with 10% vol/vol 0.9 M NaOH. The catalytic rate was calculated as moles of p-NC produced per minute, normalized to moles of enzyme used. All assays were performed in triplicate.

### 4.3 Differential Scanning Calorimetry

CYP116B5-SOX and CYP116B5-SOX-(PPII) were analyzed using a MicroCal VP-DSC instrument (Malvern, UK) over a temperature range of 25–90 °C, at a scan rate of 90 °C/h, with a 10 min of prescan equilibration. All protein samples were prepared in 50 mM KPI buffer at pH 6.8 with 1% glycerol. All samples were at an enzyme concentration of 0.6 mg/mL.^3,44^

### 4.4 Residual Activity

Activity trials were conducted using sarcosine to drive the catalysis of CYP116B5-SOX­(PPII) and CYP116B5-SOX at a final concentration of 250 mM, while 1 mM H_2_O_2_ was directly added for CYP116B5-hd. All reactions were performed in 50 mM KPi buffer at pH 6.8 supplemented with 1.2 mM *p*-NP as the CYP116B5 substrate. Each enzyme was used at a final concentration of 0.25 μM. Enzymes were preincubated in 50 mM KPi at pH 6.8 for 20 min at various temperatures: 30 °C, 35 °C, 40 °C, 42.5 °C, 45 °C, 50 °C, 55 °C, and 60 °C. Subsequently, the enzymes were diluted in the reaction solution containing either sarcosine or hydrogen peroxide. Samples were maintained at 30 °C for 15 min. To terminate the reaction, 1% v/v trichloroacetic acid (TCA) was added to each sample, and the amount of p-NC produced was measured as described in this work. All assays were performed in triplicate. Experimental data fitting was performed using a four-parameter Hill-like equation:

$$f=\frac{y_{0}+aT^{b}}{{T_{50}}^{b}+T^{b}}$$

*T*_50_ and *b* parameters were used to define the temperature-dependent stability of the three enzymes.^30^

### 4.5 H_2_O_2_ Production

The production of H_2_O_2_ was measured by exploiting the HRP-coupled reaction as previously described.^15,24,32^ Protein samples of CYP116B5-SOX or CYP116B5-SOX­(PPII) were mixed at a final concentration of 5 nM to a mixture containing 0.5 μM horseradish peroxidase (HRP) and 250 μM ABTS in 50 mM KPi at pH 6.8. The reaction was started by the addition of 5 mM sarcosine and the absorbance at 420 nm was monitored for 1200 s. The temperature was controlled using a Peltier Agilent 89090 set at 30 °C. Spectra were recorded every 5 s using an Agilent 8453 UV–Vis spectrophotometer. The initial rate of ABTS•^+^ formation was measured by monitoring the absorbance at 419 nm after 200 s. The concentration of hydrogen peroxide was calculated on the basis of a calibration curve. The calibration curve was obtained by monitoring the absorbance at 419 nm over 1200 s of samples containing 0, 10, 25, 50, 75, or 100 μM H_2_O_2_, 0.5 μM HRP, and 250 μM ABTS in 50 mM KPi at pH 6.8. To analyze the residual activity of the enzymes, protein samples were incubated in KPi solution at pH 6.8 at 45 or 60 °C for 20 min, and the activity was measured using the same method. All experiments were performed in triplicate.

### 4.6 ATR-FTIR Experiments

FTIR analyses were performed using a previously reported procedure,^10,31^ with modifications. Experiments were conducted at room temperature on a Bruker Tensor 27 FT-IR spectrophotometer (Bruker Instruments, USA) using an ATR accessory (Harrick Scientific Products, USA) with a scan velocity of 10 kHz and a resolution of 4 cm^–1^. A 15 μL of 80–100 μM protein sample was deposited as a thin film directly on ATR germanium reflection crystal. Spectra were collected between 4000 and 800 cm^–1^ wavenumbers. During acquisition, the spectrophotometer sample chamber was continuously purged with nitrogen. For H/D exchange kinetics, nitrogen gas was enriched with ^2^H_2_O by bubbling it through a bottle containing 200 mL of ^2^H_2_O. 140 scans were recorded and averaged every 2 min over a 24 min period. The H/D exchange process was monitored by following the changes in the Amide II band. At each time point, spectra were collected in triplicate and averaged using the Opus software (Bruker Instruments, Billerica, MA). Spectra were carefully analyzed by subtraction of buffer samples acquired under the same conditions and corrected to the Amide I band. H/D exchange kinetics was studied by fitting the amide II relative intensity change as a function of time using SigmaPlot software. H/D-exchange kinetic parameters were extrapolated by fitting the curve of the Amide II intensity change to the sum of two exponential equations:

$$f1=ae^{-k1x};\;f2=F_{0}+ae^{-k2x}$$

### 4.7 Substrate Screening

The P450 116B5 activity was tested using CYP116B5-hd toward a library of small alkyl and aryl compounds, including linalool, ethyl levulinate, indole, indene, naphthalene, tetralin, styrene, S-limonene, and R-limonene. The enzyme was diluted in 50 mM KPi, pH 6.8, to a final concentration of 10 μM, with 1 mM of the given compound and of 1 mM H_2_O_2_ and incubated at 28 °C for 3 h. Samples were extracted by adding a 1:1 volume of methyl *tert*-butyl ether (MTBE) and collecting the organic phase after centrifugation at 15,000*g*. Qualitative analysis of the reaction products was performed on an Agilent 7890 with a flame ionization detector (FID) using a (5%-phenyl)-methylpolysiloxane nonpolar Agilent J&W HP-5 column (30 m, 0.32 mm, 0.50 μm). Gas chromatography methods were adapted from previous literature for linalool,^45^ ethyl levulinate,^46^ R-/S-limonene,^47^ naphthalene, tetralin, indene and indole,^48^ and styrene.^49^

### 4.8 Styrene Reactions

The kinetics study was performed using purified CYP116B5-SOX­(PPII) diluted to a final concentration of 3 μM in 50 mM KPi at pH 6.8 with 10% ethanol. CYP116B5-SOX­(PPII) reactions in the presence of 0.12, 0.25, 0.5, 1, 2, 4, 8, 12, or 18 mM styrene were initiated by adding 100 mM sarcosine and carried out for 20 min at room temperature. A control experiment was performed by adding 1 mM styrene to either CYP116B5-SOX­(PPII) or CYP116B5-hd. Cell-free extract reactions were performed using the same *E. coli* strain batch transformed with pET-28a­(CYP116B5-SOX­(PPII)) that was used to purify the protein. Cell lysis and extraction were performed following the same method described in Methods section. Reactions were initiated by adding 250 mM sarcosine or 1, 5, or 10 mM H_2_O_2_. Control samples were prepared in the same way without sarcosine or styrene, respectively. Samples were extracted by adding a 10× volume of methyl *tert*-butyl ether (MTBE) and centrifuged for 10 min at 13,500*g*. The organic phase was collected and analyzed by GC-MS using a Shimadzu single quadrupole GCMS-QP2020 NX equipped with a SH-I5MS capillary column. Helium was used as the carrier gas with a flow rate of 1.17 mL min^–1^ and a split ratio of 10. The injector temperature was set to 250 °C. The oven temperature was set to 40 °C for 2 min, then a first temperature ramp of 40–180 °C was applied at 10 °C min^–1^. A second temperature ramp of 180–280 °C was applied at 40 °C min^–1^ and the temperature was held for 1 min. Mass spectra were collected using electrospray ionization in single-ion monitoring (SIM) mode (*m*/*z*: 91, 104, 132).

## Abbreviations

SOX: monomeric sarcosine oxidase
PPII: polyproline type II helix
CYP116B5-hd: CYP116B5 heme domain
CYP116B5-fl: CYP116B5 full length
DSC: Differential Scanning Calorimetry
ATR-FTIR: Attenuated Total Reflectance Fourier Transform Infrared
HRP: horseradish peroxidase
*p*-NP: para-nitrophenol
*p*-NC: para-nitrocatechol
